# Representation of Women and Underrepresented Groups in US Academic Medicine by Specialty

**DOI:** 10.1001/jamanetworkopen.2021.23512

**Published:** 2021-08-30

**Authors:** Alexander Yoo, Benjamin P. George, Peggy Auinger, Emma Strawderman, David A. Paul

**Affiliations:** 1 Department of Neurology, University of Rochester Medical Center, Rochester, New York; 2 Department of Brain and Cognitive Sciences, University of Rochester, Rochester, New York; 3 Department of Neurosurgery, University of Rochester Medical Center, Rochester, New York

## Abstract

This cross-sectional study compares trends in representation of women and underrepresented groups in medicine in US academic medicine by specialty.

## Introduction

Diversity in the physician workforce has always been and remains a critical issue. Prior studies demonstrate the number of women and members of underrepresented groups in medicine (URM), such as American Indian or Alaskan Native, Black, Latino or Hispanic, and Native Hawaiian or other Pacific Islander individuals, are increasing throughout academic medicine.^1,2^ However, little is known in the current literature regarding variation and trends in demographics of academic faculty across medical specialties or the retention of residents identifying as women or URM as academic faculty. This study adds to the literature by examining 30-year demographic trends across academic medicine departments and providing novel comparisons of the proportion of individuals identifying as women or URM between academic faculty and specialty-matched residents.

## Methods

This cross-sectional study was deemed exempt from review and informed consent by the University of Rochester institutional review board because it was deemed non–human participant research. This study is reported following the Strengthening the Reporting of Observational Studies in Epidemiology (STROBE) reporting guideline.

We evaluated the distribution of women and URM among US medical school faculty for 16 clinical academic medicine departments using the Association of American Medical Colleges Faculty Roster from 1990 through 2019. Race/ethnicity of faculty was self-identified. Linear mixed-effects models were used to estimate the mean change per year (ie, linear slope) in percentages of women and percentages of URM, which included department, time, and a department-by-time interaction term with an autoregressive correlation structure for repeated measures (eAppendix in the Supplement). Bonferroni correction for multiple comparisons was used; statistical significance defined as 2-sided *P* < .003.

Demographics, including race and ethnicity, of US resident physicians from 2012 to 2013 were obtained from the Accreditation Council of Graduate Medical Education. Representation ratios^3^ were calculated by dividing the proportion of women or URM faculty in 2019 by the proportion of women or URM residents in the 2012 to 2013 academic year (allowing for a 6-year time lag). This metric denotes the representativeness of women or URM faculty compared to the corresponding trainee pipeline (eAppendix in the Supplement).

Analyses were performed using SAS version 94 (SAS Institute), and representation ratios were calculated using R version 4.04 (R Project for Statistical Computing). Data were analyzed in December 2019.

## Results

From 1990 to 2019, there were a total of 3 146 342 faculty entries, including 1 089 892 women (34.6%) and 2 252 134 faculty entries for White physicians (71.9%). Proportions of women faculty increased, with women comprising more than 50% of faculty members in 5 of 16 clinical academic departments by 2019 (Table). Proportions of URM faculty also increased for 8 of 16 specialties (Table).

**Table.** Women and Underrepresented Groups in US Academic Medical School Faculty, 1990-2019^a^

Academic department	Total faculty, No. (N = 3 146 342)		Women			Underrepresented in medicine^b^		
	1990	2019	Faculty, No. (%)		Linear slope (95% Cl)^c^^,^^d^	Faculty, No. (%)		Linear slope (95% Cl)^c^
			1990	2019		1990	2019	
Anesthesiology	3397	9248	822 (24.2)	3361 (36.3)	0.43 (0.33 to 0.53)	196 (5.8)	923 (10.0)	0.15 (0.05 to 0.24)^e^
Dermatology	469	1548	122 (26.0)	814 (52.6)	0.91 (0.81 to 1.01)	30 (6.4)	116 (7.5)	0.04 (−0.05 to 0.13)
Emergency medicine	214	5910	35 (16.4)	2251 (38.1)	0.74 (0.64 to 0.84)	12 (5.6)	622 (10.5)	0.17 (0.08 to 0.26)^e^
Family medicine	2132	5923	604 (28.3)	3152 (53.2)	0.85 (0.75 to 0.95)	174 (8.2)	821 (13.9)	0.20 (0.10 to 0.29)^e^
Internal medicine	16 606	44 721	3125 (18.8)	18 414 (41.2)	0.77 (0.67 to 0.87)	892 (5.4)	4408 (9.9)	0.15 (0.06 to 0.25)^e^
Neurology	2108	6312	398 (18.9)	2633 (41.7)	0.77 (0.67 to 0.87)	75 (3.6)	495 (7.8)	0.15 (0.05 to 0.24)^e^
OB/GYN	2950	6698	840 (28.5)	4389 (65.5)	1.27 (1.17 to 1.37)	293 (9.9)	1081 (16.1)	0.21 (0.12 to 0.31)^e^
Ophthalmology	1321	3151	232 (17.6)	1277 (40.5)	0.77 (0.67 to 0.87)	51 (3.9)	217 (6.9)	0.10 (0.01 to 0.20)
Orthopedics	1081	4335	93 (8.6)	862 (19.9)	0.39 (0.29 to 0.49)	39 (3.6)	268 (6.2)	0.09 (−0.004 to 0.18)
Otolaryngology	743	2312	131 (17.6)	813 (35.2)	0.60 (0.50 to 0.70)	15 (2.0)	168 (7.3)	0.18 (0.09 to 0.27)^e^
Pathology-clinical	2248	4709	540 (24.0)	2041 (43.3)	0.67 (0.57 to 0.77)	113 (5.0)	344 (7.3)	0.08 (−0.01 to 0.17)
Pediatrics	7479	24 145	2695 (36.0)	14 375 (59.5)	0.80 (0.70 to 0.90)	510 (6.8)	2732 (11.3)	0.16 (0.06 to 0.25)^e^
Physical medicine and rehabilitation	704	1847	271 (38.5)	901 (48.8)	0.35 (0.25 to 0.45)	45 (6.4)	183 (9.9)	0.12 (0.03 to 0.21)
Psychiatry	6259	11 500	1716 (27.3)	6246 (54.3)	0.92 (0.82 to 1.02)	417 (6.6)	1169 (10.2)	0.12 (0.03 to 0.21)
Radiology	4662	10 118	874 (18.7)	2999 (29.6)	0.38 (0.28 to 0.48)	242 (5.2)	685 (6.8)	0.05 (−0.04 to 0.15)
Surgery	6158	16 546	562 (9.1)	4458 (26.9)	0.61 (0.51 to 0.71)	349 (5.7)	1551 (9.4)	0.13 (0.04 to 0.22)
Total	58 567	159 023	13 060 (22.3)	68 986 (43.4)	0.70 (0.68 to 0.73)	3453 (5.9)	15 783 (9.9)	0.13 (0.11 to 0.15)^e^

Abbreviation: OB/GYN, obstetrics and gynecology.

^a^ Data are from the Association of American Medical Colleges Faculty Roster as of December 31, 2019.

^b^ Underrepresented groups include American Indian or Alaskan Native, Black, Hispanic, Latino or of Spanish origin, Native Hawaiian or other Pacific Islander, and multiple races with Hispanic ethnicity individuals. Individuals identifying as multiple races and non-Hispanic identity were not included owing to the inability to further distinguish subcategories of race and ethnicity.

^c^ Linear mixed-effects models were used to estimate the mean change per year in percentages of women and percentages of underrepresented in medicine individuals and included department, time, and a department by time interaction, with an autoregressive AR(1) correlation structure for the repeated measures.

^d^ *P* value comparing slope to zero was < .003 for all comparisons.

^e^ *P* value comparing slope to zero  < .003.

In 2019, specialties with high proportions of women faculty did not necessarily have high representativeness compared with residents. Obstetrics and gynecology, which has the highest proportion of women faculty, demonstrated the third lowest representation ratio (0.81). In contrast, despite having the lowest overall proportion of women faculty, orthopedic surgery had the highest representation ratio (1.48) (Figure, A). In respect to URM, most specialties had representation ratios less than 1.0 (overall representation ratio, 0.76) (Figure, B-D).

**Figure.**
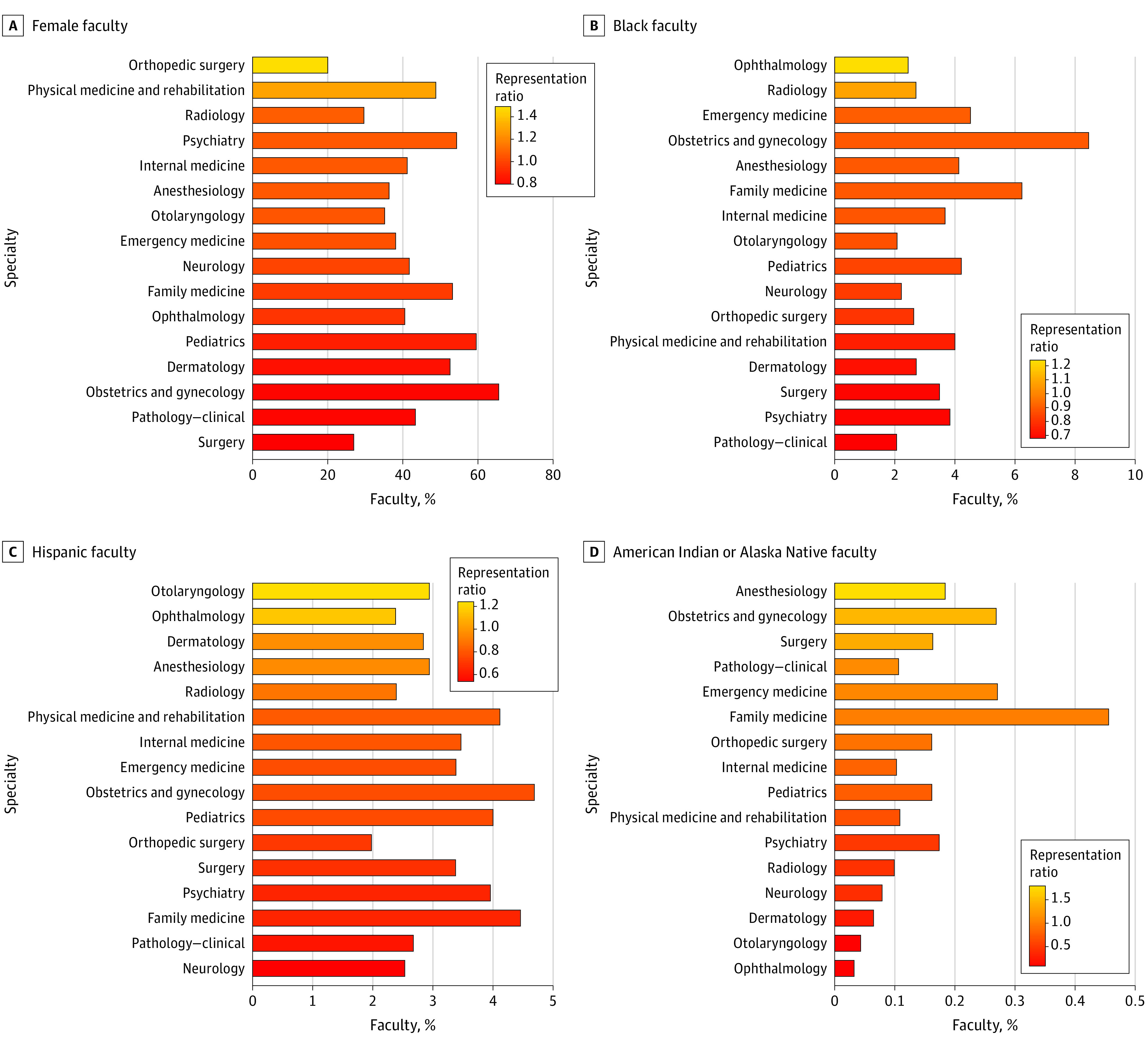
Representation Ratio and Percentage of Women and Underrepresented Groups in Medicine Faculty in US Medical Schools by Academic Department Data are from the Association of American Medical Colleges Faculty Roster as of December 31, 2019, and Accreditation Council of Graduate Medical Education for 2012 to 2013. Representation ratio is the proportion of women or underrepresented group in medicine for faculty in 2019 divided by that for residents 6 years prior, in the 2012 to 2013 academic year. Academic medical departments are displayed by magnitude of representation ratio. Underrepresented groups depicted include Black, Hispanic, and American Indian or Alaska Native individuals. Individuals identified as multiple race, Native Hawaiian, or Other Pacific Islander were not included owing to the inability to distinguish these subcategories within resident data.

## Discussion

This cross-sectional study found increases in the proportions of women faculty across clinical academic departments over the past 3 decades. Racial and ethnic diversity among faculty also increased, although at a lower rate. Increasing faculty diversity can be partially attributed to comparably modest improvements in diversity among medical students and residents.^4^ However, URM faculty are underrepresented compared with the resident pipelines for most specialties. Nearly all departments captured only a fraction of the available URM resident pipeline, and there were differences in representation ratios across departments for women. Further investigation is needed to understand factors that may dissuade or obstruct women and URM trainees from pursuing academic careers. Previous studies, such as a 2013 study by Peek et al,^5^ have found that medical schools with URM role models and available, experienced mentors (URM and non-URM) were more likely to have high proportions of URM students. Studies are needed to determine modifiable differences and how to implement change to optimize faculty demographics.

This study has some limitations. The interpretation of the representation ratio in this study is limited by the inability to control for trainee preferences for academics and does not account for personal and structural factors that may influence career choice. Other limitations include the use of nationally aggregated deidentified data, which prohibits control of confounding factors, including regional demographics and status of Historically Black Colleges and Universities. Further study of individualized faculty and institutional-level data are needed.

## Notes

Supplement.
eMethods.
