# Supplementary material for: Representation of Women and Underrepresented Groups in US Academic Medicine by Specialty
Source: JAMA Netw Open. 2021 Aug 30;4(8):e2123512. doi: 10.1001/jamanetworkopen.2021.23512 (PMC8406079; doi:10.1001/jamanetworkopen.2021.23512)
Supplement: Supplement. — eMethods. [file jamanetwopen-e2123512-s001.pdf]

Supplemental Online Content

Yoo A, George BP, Auinger P, Strawderman E, Paul DA. Representation of women and underrepresented groups in US academic medicine by specialty. *JAMA Netw Open*. 2021;4(8):e2123512. doi:10.1001/jamanetworkopen.2021.23512

eMethods.

This supplemental material has been provided by the authors to give readers additional information about their work.

© 2021 Yoo A et al. *JAMA Network Open.*

eMethods

Study Design

This is a retrospective observational cross-sectional study of the Association of American Medical Colleges (AAMC) Faculty Roster from 1990 through 2019[1] and Accreditation Council of Graduate Medical Education (ACGME) Data Resource Book, 2012-2013[2] to examine differences in faculty diversity across various academic clinical specialties. Data are aggregate for all US academic institutions stratified by sex, race/ethnicity, and academic department or specialty. Aggregate data are publicly available online or upon request from the respective data sources. Given that data used in the study are deidentified and aggregated on the national level, the University of Rochester Medical Center Research Subjects Review Board deemed the study exempt from federal regulations for the protection of human research participants.

Data Source

The AAMC initiated the Faculty Roster in 1966 to support national policy studies by collecting comprehensive information on the characteristics of academic faculty members at LCME accredited US medical schools. The AAMC Faculty Roster is available as aggregate data from all accredited US MD-granting medical schools on all full-time faculty as of December 31st in each respective year of the data stratified by sex, race/ethnicity, and department classification. The Faculty Roster utilizes the official name given to each department by each medical school. This study included n=16 clinical departments as reported by the AAMC: Anesthesiology, Dermatology, Emergency Medicine, Family Medicine, Internal Medicine, Neurology, Obstetrics & Gynecology, Ophthalmology, Orthopedic Surgery, Otolaryngology, Clinical Pathology,

© 2021 Yoo A et al. *JAMA Network Open.*

Pediatrics, Physical Medicine & Rehabilitation, Psychiatry, Radiology, and Surgery. These departments included n=3,146,342 faculty from 1990 to 2019, or n=58,567 from 124 US medical schools in 1990 and n=159,023 from 149 US medical schools in 2019. Departments excluded from the analysis were Public Health & Preventive Medicine, Social Sciences, Dentistry, Veterinary Sciences, Other Health Professions, Anatomy, Biochemistry, Microbiology, Basic Science Pathology, Pharmacology, Physiology, Other Basic Sciences, and All Others.

The ACGME Data Resource Book is collected from the Accreditation Data System, a web-based software system collected on the program level. The Data Resource Book contains the most recent data on ACGME accredited institutions and programs and represents a cross-section as of June 30th in the respective year of the data (i.e., 2012-2013). The ACGME report specialty and subspecialty by year. ACGME classified specialties were matched to included AAMC reported departments. Subspecialties (e.g., Child Neurology) and departments not reported in the AAMC Faculty Roster (e.g., Radiation Oncology) were excluded from the analysis. The included specialties represent n=89,414 residents in the 2012-2013 academic year.

All data were received from the data source in nationally aggregated data stratified by sex, race/ethnicity, and academic department or specialty and are publicly available online, or upon request.

Race/Ethnicity Classifications

Underrepresented groups in medicine (URM) were defined as American Indian or Alaskan Native, Black or African American, Hispanic, Latino or of Spanish Origin, Native Hawaiian or Other Pacific Islander, and Multiple Race – Hispanic, consistent with prior

© 2021 Yoo A et al. *JAMA Network Open.*

reports.[3] Where comparisons were made to the resident population, URM was collectively defined as Black, Hispanic, and American Indian or Alaskan Native, with the exclusion of mixed race categories, Native Hawaiian, and Pacific Islander due to the limited reporting of corresponding resident race/ethnicity in the ACGME Data Resource Book; however, these groups comprised ~3% of faculty data.

Outcomes

This study examines trends in the percentage of URM and women within clinical academic faculty stratified by specialty from 1990 to 2019.

The study also examines sex and race/ethnicity representation within academic faculty compared to the pool of available residents. The metric used is referred to as the "representation ratio".[4] The representation ratio was calculated by dividing the proportion of women or URM faculty in 2019 by the proportion of women or URM residents in the 2012-2013 academic year. A six-year time lag between faculty and resident populations was examined to account for the diffusion of residents into faculty positions. This metric denotes the representativeness of women or URM faculty in academic departments compared to the corresponding specialty trainee pipeline. For example, representation ratio of 1.0 indicates a perfect match in the proportions of faculty compared to residents, whereas representation ratio values below 1.0 reflect faculty underrepresentation compared to residents.

Statistical Analysis

Linear mixed-effects models were used to estimate the mean change per year (linear slope) in percent women and percent URM and included department, time (1990-2019), and a department by time interaction, with an autoregressive AR(1) correlation

© 2021 Yoo A et al. *JAMA Network Open.*

structure for the repeated measures. Each estimated departmental slope was compared to zero and to the slope of the other departments combined. A Bonferroni correction was applied to account for multiple comparisons and $p<0.003$ was considered statistically significant. SAS version 9.4 (SAS Institute, Cary, NC) was used for analyses. The **Figure** reporting Representation Ratios was developed using R version 4.04 (R Core Team [2017]). This study was conducted in compliance with STROBE reporting guidelines for cross-sectional studies.

Limitations

There are several limitations to the study. The interpretation of the representation ratio in this study is limited by the inability to control for trainee preferences regarding academic positions and does not account for personal and structural factors that may influence career choice. Furthermore, the study used nationally aggregated de-identified data which restricts the granularity of the analysis and prohibits control of potentially confounding factors such as geographic location, regional demographics, and status of Historically Black Colleges and Universities – which tend toward greater diversity. Furthermore, due to differences in reporting of race/ethnicity and department classification between the AAMC Faculty Roster and ACGME Data Resource Book comparisons between faculty and resident populations may be imperfect. This study is also restricted to MD-granting (allopathic) institutions by nature of the data source and does not represent the academic faculty population for Osteopathic medical schools. Further study of individualized faculty and institutional data with appropriate control of regional demographics is needed.

© 2021 Yoo A et al. *JAMA Network Open.*

Supplementary References

1) Association of American Medical Colleges. Faculty Roster: U.S. Medical School Faculty 1990-2019. Accessed November 25, 2020. https://www.aamc.org/data-reports/faculty-institutions/report/faculty-roster-us-medical-school-faculty

2) Accreditation Council for Graduate Medical Education. ACGME Data Resource Book 2012-2013. Accessed January 12, 2021. https://www.acgme.org/about-us/publications-and-resources/graduate-medical-education-data-resource-book/

3) Ogunwole SM, Dill M, Jones K, Golden SH. Trends in Internal Medicine Faculty by Sex and Race/Ethnicity, 1980-2018. *JAMA Netw Open.* 2020;3(9):e2015205.

4) Hofler LG, Hacker MR, Dodge LE, Schutzberg R, Ricciotti HA. Comparison of Women in Department Leadership in Obstetrics and Gynecology With Those in Other Specialties. *Obstet Gynecol*. 2016;127(3):442-447.

© 2021 Yoo A et al. *JAMA Network Open.*
